# Supplementary material for: Is Osteopontin a Good Marker for Bone Metastasis in Canine Mammary Gland Tumor and Prostate Cancer?
Source: Animals (Basel). 2023 Oct 14;13(20):3211. doi: 10.3390/ani13203211 (PMC10603680; doi:10.3390/ani13203211)
Supplement: Supplementary file 1 [file animals-13-03211-s001.zip › Supplementary Table S2.pdf]

Table S2. Regression logistic multiple analysis with each variable individually as dependent variable and the others as independent variables.

Weight as dependent variable

Significant different than zero?	Variable	|t|	P value	P value summary
β0	Intercept	1,388	0,1797	ns
β1	B: Age	0,172	0,8651	ns
β2	C: Histological Subtype	0,04181	0,967	ns
β3	E: Disease free-interval	1,352	0,1908	ns
β4	F: Overall survival	0,9122	0,372	ns
β5	G: OPN expression	0,4817	0,635	ns
β6	H: Metastasis	1,093	0,2868	ns

Age as dependent variable

Significant different than zero?	Variable	|t|	P value	P value summary
β0	Intercept	0,3664	0,7177	ns
β1	A: Weight	0,172	0,8651	ns
β2	C: Histological Subtype	2,774	0,0114	*
β3	E: Disease free-interval	0,9183	0,3689	ns
β4	F: Overall survival	0,1085	0,9146	ns
β5	G: OPN expression	0,9637	0,3462	ns
β6	H: Metastasis	0,3199	0,7522	ns

Histological subtypes as dependent variable

Significant different than zero?	Variable	|t|	P value	P value summary
β0	Intercept	2,168	0,0418	*
β1	A: Weight	0,04181	0,967	ns
β2	B: Age	2,774	0,0114	*
β3	E: Disease free-interval	0,6641	0,5138	ns
β4	F: Overall survival	2,372	0,0273	*
β5	G: OPN expression	0,484	0,6334	ns
β6	H: Metastasis	0,3596	0,7227	ns

Disease-free interval as dependent variable

Significant different than zero?	Variable	|t|	P value	P value summary
β0	Intercept	0,3841	0,7048	ns
β1	A: Weight	1,352	0,1908	ns
β2	B: Age	0,9183	0,3689	ns
β3	C: Histological Subtype	0,6641	0,5138	ns
β4	F: Overall survival	3,234	0,004	**
β5	G: OPN expression	0,7856	0,4409	ns
β6	H: Metastasis	0,3886	0,7015	ns

Overall survival as dependent variable

Significant different than zero?	Variable	|t|	P value	P value summary
β0	Intercept	2,129	0,0452	*
β1	A: Weight	0,9122	0,372	ns
β2	B: Age	0,1085	0,9146	ns
β3	C: Histological Subtype	2,372	0,0273	*
β4	E: Disease free-interval	3,234	0,004	**
β5	G: OPN expression	0,9711	0,3425	ns
β6	H: Metastasis	0,6078	0,5499	ns

Osteopontin as dependent variable

Significant different than zero?	Variable	|t|	P value	P value summary
β0	Intercept	7,002	<0,0001	****
β1	A: Weight	0,4817	0,635	ns
β2	B: Age	0,9637	0,3462	ns
β3	C: Histological Subtype	0,484	0,6334	ns
β4	E: Disease free-interval	0,7856	0,4409	ns
β5	F: Overall survival	0,9711	0,3425	ns
β6	H: Metastasis	12,37	<0,0001	****

Metastasis as dependent variable

Significant different than zero?	Variable	|t|	P value	P value summary
β0	Intercept	8,559	<0,0001	****
β1	A: Weight	1,093	0,2868	ns
β2	B: Age	0,3199	0,7522	ns
β3	C: Histological Subtype	0,3596	0,7227	ns
β4	E: Disease free-interval	0,3886	0,7015	ns
β5	F: Overall survival	0,6078	0,5499	ns
β6	G: OPN expression	12,37	<0,0001	****

ns: not statistically different; ***: statistical difference.
